# How to convene an international health or development commission: ten key steps

**DOI:** 10.1093/heapol/czx179

**Published:** 2018-01-10

**Authors:** Gavin Yamey, Lawrence H Summers, Dean T Jamison, Jessica Brinton

**Affiliations:** 1 Duke Global Health Institute, Center for Policy Impact in Global Health, 310 Trent Drive, Durham, NC 27708,; 2 Duke Sanford School of Public Policy, 201 Science Dr, Durham, NC 27708,; 3 Harvard University, Harvard Kennedy School, Mossavar-Rahmani Center for Business and Government, 79 John F. Kennedy St, Cambridge, MA 02138,; 4 Institute for Global Health Sciences, University of California, San Francisco, San Francisco, CA, USA and; 5 Bill & Melinda Gates Foundation, Seattle, WA, USA

**Keywords:** Policy, policy implementation, policy process, health care, priority setting

## Abstract

The Commission on Investing in Health (CIH), an international group of 25 economists and global health experts, published its *Global Health 2035* report in *The Lancet* in December 2013. The report laid out an ambitious investment framework for achieving a “grand convergence” in health—a universal reduction in deaths from infectious diseases and maternal and child health conditions—within a generation. This article captures ten key elements that the CIH found important to its process and successful outcomes. The elements are presented in chronological order, from inception to post-publication activities. The starting point is to identify the gap that a new commission could help to narrow. A critical early step is to choose a chair who can help to set the agenda, motivate the commissioners, frame the commission’s analytic work, and run the commission meetings in an effective way. In selecting commissioners, important considerations are their technical expertise, ensuring diversity of people and viewpoints, and the connections that commissioners have with the intended policy audience. Financial and human resources need to be secured, typically from universities, foundations, and development agencies. It is important to set a clear end date, so that the commission’s work program, the timing of its meetings and its interim deadlines can be established. In-person meetings are usually a more effective mechanism than conference calls for gaining commissioners’ inputs, surfacing important debates, and ‘reality testing’ the commission’s key findings and messages. To have policy impact, the commission report should ideally say something new and unexpected and should have simple messages. Generating new empirical data and including forward-looking recommendations can also help galvanize policy action. Finally, the lifespan of a commission can be extended if it lays the foundation for a research agenda that is then taken up after the commission report is published.

Key Messages

- High-level commissions on global health or development can be influential in raising the profile of a neglected topic, in stimulating discussion and debate and in shaping policy.
- A commission report is more likely to have an impact if it disrupts orthodoxies and challenges readers, pushing them to think in new ways.
- Other factors that are likely to affect whether a commission report gets noticed include the novelty, simplicity and timing of the messaging and the inclusion of forward-looking recommendations.
- It can also be helpful, prior to publishing the report, to develop policy engagement and outreach plans that aim to reach policy-relevant audiences through different channels (e.g. the media, personal networks and online and social media platforms).

## Introduction

In December 2016, we held the final meeting of the Commission on Investing in Health (CIH), an international commission of 25 economists and global health experts from both the global north and south. Over the last 4 years, this group has been engaged in making the economic case for investing in health at a variety of international venues, including United Nations agencies, development banks, multilateral and bilateral development agencies and ministries of health and finance.

The Commission, which began in 2012, was prompted by the upcoming 20th anniversary of the 1993 World Development Report (WDR 1993), called *Investing in Health* (World Bank 1993). Bringing together experts and new data, the CIH re-examined the economic case for investing in health and took the previous report a step further by developing a roadmap for achieving large gains in global health within a generation. The CIH’s final report, *Global Health 2035*, published in *The Lancet* in December 2013, (Jamison *et al.* 2013) showed that with scaled-up investments in delivering health interventions and developing new health technologies, a ‘grand convergence’ in global health could be achieved by 2035. We defined this new concept of a grand convergence as a reduction in premature deaths from infectious disease and maternal and child health conditions down to universally low levels. *Global Health 2035* was used as an argument for continued and increased funding and programmatic support for global health among bilateral and multilateral donors and country governments.

Since publication of the report, organizations or individuals who are launching their own commissions have reached out to the CIH, and its secretariat, to ask us to share our reflections on what worked well—and not so well—in convening the CIH. For example, we recently shared our thoughts with the chairs of three forthcoming *Lancet* commissions—on global tuberculosis, global health and the law, (Gostin *et al.* 2015) and on reframing non-communicable diseases and injuries for the poorest billion (Bukhman *et al.* 2015). This article was prompted by these requests. It represents our best effort to capture the key elements that we found important to our commission process and successful outcomes. The article was written by the CIH chair (L.H.S.), co-chair (D.T.J.), lead writer (G.Y.) and an author from one of the five funders of the CIH (J.B.).

We present our suggestions in chronological order, taking readers through ten key steps from inception to post-publication policy activities. By adopting this stepwise approach, we do not wish to suggest that convening a commission is a simple, ‘mechanical’ process. It is also an inherently political process—politics are at play, for example, when it comes to choosing the topic, deciding on which commissioners to invite and planning the launch and policy outreach activities. Shiffman has previously highlighted the political nature of *Lancet* commissions, asking: ‘What are the benefits and disadvantages of a medical journal undertaking such activities in the global health field? Which issues are more likely to receive a hearing, and which may be neglected? Whose voices are privileged by this phenomenon? Whose are missed? How is the power of other global health actors enhanced or diminished, including researchers in medical and public health schools, the WHO, the World Bank, the Global Fund and civil society movements (Shiffman 2014)?’ Throughout our article, we comment on some of these political aspects of our commission.

## Key steps in convening a commission and producing a high-impact report

### Identifying the gap that the commission could help to narrow

The starting point for any new commission on global health or development is to clearly identify and articulate why such a commission is needed. What gap in the health or development policy landscape will it help to narrow? Commissions are more likely to be successful—as defined by having an influence on policy—if they help to narrow or close an identifiable and important gap.

Dean Jamison initiated the CIH because he saw two critical policy gaps that he believed a *Lancet* commission could help to address. First, he identified a clear need to revisit the arguments for investing in health first proposed in WDR 1993. In the two decades after publication of WDR 1993, there had been tremendous scientific advances, such as the advent of highly active antiretroviral therapy and long-lasting insecticidal bed nets, as well as institutional advances, such as the launch of multilateral financing programs like the Global Fund to Fight AIDS, Tuberculosis and Malaria. The gains in health over these two decades were unexpectedly large—much larger than the gains that the authors of WDR 1993 thought possible. In the closing era of the Millennium Development Goals (MDGs) and the opening era of the Sustainable Development Goals (SDGs), when the value of targeted investments in health was being debated and scrutinized, (Lancet 2012) a commission was needed to revisit the evidence on the health and economic returns to investing in health.

Alongside the widespread questioning of whether it was time to ‘move on’ from investing in health and to invest instead in other development sectors, (Lancet 2012) Jamison was also alarmed by the widespread questioning of whether governments should really be playing a central role in financing health. A second gap that the commission aimed to fill was to show to policymakers the strong and compelling evidence of the value of public financing of health, not just for improving health but also for protecting the poor from the financial ruin caused by out-of-pocket medical expenses (Xu *et al.* 2007) and for curbing unproductive cost escalation (Clements *et al.* 2012).

### Choosing the commission chair

One of the earliest, and arguably most important, decisions that needs to be made is who will chair the commission. As mentioned, this is one of the steps that is influenced by politics. The choice matters because engaged chairs will set the agenda, inspire and motivate the commissioners, frame the analytic work and run the commission meetings in ways that get the most out of the discussions. They will also leverage their personal and policy connections when it comes to policy engagement and outreach. When a commission is first announced, typically through an editorial in the journal that will later be publishing the commission’s final report (Bukhman *et al.* 2015 is an example of such an editorial), the choice of chair will get noticed and scrutinized. The choice sends a message about the underlying politics of the commission.

Announcing a chair who has an international reputation and presence, and is known as a key opinion leader, as we did with Larry Summers and Dean Jamison for the CIH, can set the right tone and expectations, signalling that the commission itself will be high level and ambitious. Furthermore, there are benefits in choosing a chair that might be outside the ‘usual suspects’. In our case, Larry Summers was *not* a global health ‘insider’, which resulted in him coming to the topic with a different perspective. This ‘outsider’ status also meant that during commission meetings, he was more likely to challenge and push commissioners to think in new and out-of-the-box ways. Summers also had a reputation for being outspoken, controversial and someone who has ‘embraced the role of a provocateur’ (Appelbaum 2014). While this reputation carried risks for a commission in a scholarly journal, the benefits were greater than the pitfalls—given that commissions are effective when they provoke and challenge the *status quo* (we discuss this further below).

### Securing dedicated financial and human resources

It is difficult to write a commission report and plan commission activities without financial and human resources. It is extremely helpful to have at least one dedicated person involved in the commission who has protected time set aside for writing and ideally at least a small team to assist with research and external engagement. The main contributions of the commissioners themselves will probably be provided during the commission meetings and in advocating for the recommendations and research findings in the public sphere. Between meetings, while commissioners may have time to review early drafts or perhaps contribute a short section, it is unlikely that they will have time to write the full report, do research, or plan outreach activities.

The other advantage of having dedicated staff is that there are individuals who are managing timelines, report production and background research and analytics. They are also managing the planning and logistics for commission meetings (e.g. booking flights and hotels for commissioners), launch and outreach events and conference calls. Having in particular one dedicated senior commission member to lead on writing and one or more research assistants who can help with literature reviews, data analysis and production of display items (figures, tables, graphs and boxes) will help to ensure timely high quality evidence and writing is produced.

We acknowledge that it can be challenging raising funds to support the commission, including to cover dedicated time for writing and research and the costs of the commission meetings themselves. Nevertheless, in recent years a number of universities and external funders (e.g. the Rockefeller Foundation, the Bill & Melinda Gates Foundation, the Norwegian Development Agency, the UK Department for International Development) have supported various commissions related to global health and development. Universities can be supportive by providing, for example, staff time and meeting facilities.

### Setting a clear end date and developing the commission schedule ‘backwards’ from this date

Setting a publication date for the commission report is an important early step in the commission process. With this end date in mind, the commission’s work program, the timing of its meetings and its interim deadlines can then be established. Without an endpoint as a ‘tether’, a commission risks drifting, losing its focus and momentum.

After deciding that December 2013 would be the publication date of the CIH report, we then worked ‘backwards’ from this date to create a calendar of events and key milestones, building in time for peer review at *The Lancet*. By September 2012, the commission had been established and our analytic work and draft writing kicked off. The journal gave us a very hard deadline of the end of June 2013 to submit the report, and we timed our three in-person commission meetings in line with this submission date. We held these meetings in December 2012 (at Harvard University, Cambridge, USA, where the CIH chair was based); March 2013 (in Oslo, Norway, supported by the Norwegian Agency for Development Cooperation); and July, 2013 (in Kigali, Rwanda, hosted by Commissioner Agnes Binagwaho, Rwanda’s minister of health at that time). *The Lancet* fast-tracked the peer review process, so that by the time we met for our final meeting in Kigali, we had the reviews and were able to discuss how to respond to the reviewers’ suggestions.

In choosing a publication date, it can be helpful to try to time the report around a highly relevant event in the global health or development calendar, since this can help to amplify the report’s messages and findings. With the right timing, a commission may end up being discussed at high level fora, cited by ministers or directors of development agencies, or mentioned in national or global policy proposals. Some commissions have even fed into discussions at the World Health Assembly (WHA) or have spurred WHA resolutions.

The timing of *Global Health 2035* was primarily based on the 20-year anniversary of the 1993 World Development Report. Nevertheless, we also explicitly hooked our report to the discussions that were happening in 2013 about what would replace the MDGs after their 2015 end date. In the Introduction section, we noted that the time is right to revisit the case for investment in health because ‘we are in the closing era of the Millennium Development Goals’ and ‘the global development community is debating both a new set of post-2015 sustainable development goals and the positioning of health, including universal health coverage, in such goals’. By framing the report in this way, we were able to position ourselves to contribute to discussions at the United Nations and other forums on the SDGs. Our modelling on grand convergence was relevant to discussions about what kinds of mortality reductions would be technically feasible for the 2030 SDG targets. We also used policy windows to reach specific audiences. For example, in 2015:

- We briefed the G7/G20 sherpa team on global health at the German Chancellery in Berlin on policy options for the G7 to achieve the *Global Health 2035* goals; (Commission on Investing in Health 2015)
- We participated in the discussions around the launch of the World Bank’s Global Financing Facility in Support of Every Woman, Every Child (our analysis fed into the facility’s business plan); (World Bank 2015) and
- We conducted an analysis for Sweden’s independent Expert Group for Aid Studies, which we presented in the Swedish parliament and to the Swedish International Development Cooperation Agency, on how Sweden’s aid portfolio could become aligned with *Global Health 2035* goals (Yamey *et al.* 2016).

### Selecting the commissioners

The commission chair usually leads the process of selecting commissioners. This selection matters, because commissioners have the ability to make a commission sink or swim—the right commissioners will become champions and ambassadors of the work before, during and after the launch of the report itself. To the outside world, the choice of commissioners will say a lot about the commission’s aims and what kind of report is being crafted.

For the CIH chairs, there were four key considerations when selecting the commissioners. The first and most obvious was the particular expertise that they brought to the conceptualization and writing of the report. The 25 CIH commissioners were experts from across *a range of domains* that the commission would address—including the economics and financing of health, ending the ‘unfinished agenda’ of infections and maternal and child health conditions, curbing non-communicable diseases and injuries, strengthening health systems and driving technological innovation in health. Given the strong links between health and other development sectors, it is an advantage for health commissions to include commissioners with cross-sectoral expertise.

The second was diversity of viewpoints. There is now a wealth of research from fields such as organizational psychology and sociology showing that diverse groups produce more innovative ideas than homogeneous ones (Phillips 2014). It can be particularly helpful to bring in a variety of commissioners who are likely to bring in new perspectives and different approaches. In inviting commissioners, Larry Summers was particularly interested in avoiding the type of conventional ‘group think’ that can often take over in global health fora.

The third was the diversity of people. The CIH comprised experts who were from the north and the south, men and women, early-to-mid-career experts and experienced veterans (including a Nobel Prize winner, Kenneth Arrow) and professionals who worked in different types of institutions. The CIH commissioners included senior leaders from government, health financing agencies, the WHO, ministries of health, non-governmental organizations, foundations and think tanks as well as researchers working in the policy space. The aim was to include all of the major constituencies or stakeholders that would have an interest in the report. Including two African government officials—Agnes Binagwaho and Linah Mohohlo, who was then the Governor of the Bank of Botswana—gave the CIH a much greater understanding of the day-to-day decision-making processes of a particularly important target audience, health and finance ministers. The CIH also worked hard to enroll and engage commissioners from major multilateral health agencies, including the WHO and Gavi, the Vaccine Alliance, so that our work would be relevant to the biggest issues facing these agencies.

The fourth consideration was the potential role that the commissioners could play in connecting with the intended policy audience. Commissioners can play a key role when it comes to translating or driving the commission recommendations and findings into practical policymaking. Building on the diversity of viewpoints and the diversity of the individuals themselves, Summers and Jamison also chose commission members who would be strong advocates and had the skills and interest in taking the CIH work forward and encouraging policy influence and impact. The commissioners were very well placed to spread the report’s messages both within their own networks, and to new and different networks, where their unique role, experience and individual characteristics made them a strong messenger.

Given that our report focused on the value of investing in health, we saw our primary target audience as being international development funders and ministries of finance. We made a deliberate choice to invite commissioners who were *themselves* representatives of this audience, and who could serve as ambassadors for the report’s policy messages. For example, having senior representatives from development banks as commissioners was extremely valuable in reaching bank staff. These commissioners held launch events at the banks, including at the African Development Bank in Tunis, Tunisia and the World Bank/IMF, in Washington, DC, USA.

### Using in-person meetings to maximize the use of commissioners’ time

The CIH was able to secure enough financial support to fund three in-person meetings of the whole commission, as well as a fourth meeting of a sub-group of commissioners dedicated entirely to economics and financing. Each of these meetings lasted 2 days. Holding such meetings is a major logistical undertaking, ideally taken on by the commission’s support staff (its secretariat). Nevertheless, in our experience, they can be the most effective mechanism for gaining commissioners’ inputs, surfacing important debates, and ‘reality testing’ the commission’s key findings and messages. They also gave us the opportunity to invite additional speakers with specific expertise to give inputs into the commission. In order to get the most out of these meetings, the commissioners should be well briefed ahead of time, the meetings should be tightly chaired, and there should be a clear set of action items, next steps, and deliverables when commissioners depart. We believed that conference calls with such a large group would not be a good way to move the analytic work forward.

Given that commissioners have busy schedules, we found it very helpful for the chairs, the Commissioner leading the writing (G.Y.), and the CIH secretariat to produce draft materials ahead of each meeting for the Commissioners to review. We also found that drafting an annotated outline—one with short explanations of the content that will be in each section—early in the commission process had three important advantages.

First, it helped to quickly engage the commissioners and focus their attention; the outline was something real and tangible that they could react to, give feedback on and sharpen. Second, it was a worthwhile step in the important task of building a consensus among commissioners on what should be in or out of the report. As Larry Summers said at the launch meeting of the CIH, it was very unlikely that every commissioner would be happy with every single sentence in the report, but they would need to be able to live with the final outcome. Getting their buy-in on an annotated outline was the beginning of creating a common vision. Third, it made the writing much easier and we think it led to a better-structured report.

### Saying something new and unexpected that can be distilled into simple messages

Summers had the experience early in his career of directing a part of the World Bank whose mission was to produce policy-relevant research, and the subsequent experience of being the regular target of such research when he worked in government. At the Bank, he found that there was a commonly used template for policy reports, which was as follows:

Policy X (insert topic) is immensely important in the future of global development. Policy X must always be carefully considered in the country context. There are important intersections between Policy X and parallel areas Y and Z. The World Bank strategy on Policy X must always be integrated with the strategies of others in the global development community, especially with host countries’ strategies. Policy X offers particularly important opportunities for innovation, bringing together the public sector, private sector, and non-government organizations in the approach.

This type of template is likely to lead to a safe, uncontroversial report. The risk here is that the findings and recommendations will be bland and insipid, inspiring very little in the way of policy action. Based on our experience, we believe a commission report is more likely to have an impact on policy if it is novel and disrupts orthodoxies and challenges readers, pushing them to think in new ways.

The CIH had several key messages that provoked debate and controversy or went against the grain in terms of challenging conventional thinking. For example, at the time of writing the report, a commonly held view in development circles was that after a decade of rising aid for health, it was time for other development sectors, like climate or agriculture, ‘to take centre stage’ (Lancet 2012). *Global Health 2035* was a retort to this view, showing the primacy of health investments to sustainable investment and the remarkable returns to investment—far higher than previous estimates. Using an approach to valuing mortality reductions called ‘full income’ accounting—an approach that went beyond just using GDP—we showed that the economic returns to investing in health are much greater than previously thought (we estimated that every dollar invested in achieving grand convergence over 2015–35 could return US$9–20). In an editorial commentary by Jim Kim, World Bank President, that accompanied the CIH report, Kim said that the report provided ‘proof that improvements in human survival have economic value well beyond their direct links to gross domestic product’ (Kim 2013). Another new and unexpected message of the CIH report was that convergence could mostly be funded by low- and lower-middle income countries themselves—rather than through aid—if they allocated just 1–3% of their economic growth over the next 20 years to health spending.

Along with novelty, we believe that commissions are more likely to be noticed by policymakers if they have simple messages that can be easily transmitted to key audiences. For the CIH, our top line message was: ‘with the right investments we can end premature mortality within just one generation’. This message was easy to grasp, aspirational and was timed with conversations surrounding the end of the MDGs and the crafting of the new SDGs.

While we cannot, of course, be certain, we believe that having novel, simple messaging was a key factor in the CIH report gaining traction among policymakers after it was published. Three examples of this traction are:

- As mentioned earlier, a Swedish government committee called The Expert Group for Aid Studies commissioned the CIH to conduct a policy analysis that lays out options for reforming Swedish health aid to help achieve the vision of grand convergence set out in *Global Health 2035*. This analysis has been an important input into Sweden’s aid strategy reform process (Yamey *et al.* 2014, 2016).
- The CIH was invited by the governments of Mexico and Myanmar to present the *Global Health 2035* report, and its implications for each country, at high level forums attended by ministers of health and finance.
- The Kiel Institute for the World Economy commissioned the CIH to analyse ways in which the G7 could help support *Global Health 2035* goals, (Commission on Investing in Health 2015; Yamey *et al.* 2015) and to conduct an in-person briefing for Germany’s G7/G20 Global Health Sherpa Team in preparation for the 2015 G7/G20 summits.

Other recent commissions that had a policy impact also had one or a few main take home messages that were novel and provocative. For example, the dominant message of the April 2015 report of the *Lancet* Commission on Global Surgery was simple and alarming—five billion people lack access to safe, affordable surgical and anaesthesia care when needed (Meara *et al.* 2015). This commission played a critical role in supporting the May 2015 adoption of a World Health Assembly resolution in which, ‘for the first time, governments worldwide acknowledged and recognized surgery and anaesthesia as key components of universal health coverage and health systems strengthening’ (Price *et al.* 2015).

### Conducting empirical research as part of the commission process

Commission reports on global health and development are typically based on synthesizing existing literature and making recommendations based on this synthesis. With sufficient time and resources, some commissions have in addition been able to conduct new, empirical research and to publish the results within or alongside the final report. We believe there are several benefits to using a commission to generate new, empirical data that adds to the global knowledge bank.

New research findings can be a ‘hook’ to galvanize policymakers to take action (Haynes *et al.* 2011). For example, the Commission on Education of Health Professionals for the 21st Century, published 100 years after the 1910 Flexner report on medical education worldwide, estimated global expenditures on training health professionals (Frenk *et al.* 2010). The results were disturbing: the commission research found that annual spending on such training is only about $100 billion or < 2% of total health expenditures worldwide, ‘pitifully modest for a labour-intensive and talent-driven industry’. The commission has played an important role in shaping capacity-building efforts for the health workforce in low- and middle-income countries, such as the Global Health Service Partnership (Stuart-Shor *et al.* 2017).

By conducting research, a commission can also ‘test’ hypotheses in real time. At the start of the CIH process, an early hypothesis among the commissioners was that in middle-income countries that were graduating from health aid, high levels of avertable mortality may be concentrated in ‘pockets’ of rural poverty. A rapid analysis using demographic and health survey data from 37 low- and middle-income countries did indeed show a clustering of child deaths in rural regions of middle-income countries (Fink and Hill 2013).

Given that resource mobilization is at the very top of the political agenda when it comes to the SDGs, including the health-related SDGs, our experience suggests that policymakers are particularly ‘hungry’ for research on the costs and financing of the SDGs. Several recent *Lancet* commissions—including the CIH, (Jamison *et al.* 2013) the *Lancet* Commission on Global Surgery (Meara *et al.* 2015) and the UNAIDS-*Lancet* Commission on Defeating AIDS (Piot *et al.* 2015)—have been underpinned by modelling of the costs and financing of scaling-up disease control services.

### Proposing forward-looking recommendations and developing an action plan to accompany the report

The CIH gave a great deal of thought to the recommendations section of the final report, as we wanted our commission to be as actionable and forward-looking as possible. Our report had four major messages, each representing a key benefit of investing in health: achieving convergence, reaping an economic payoff, curbing non-communicable diseases and injuries and providing health and financial protection through pro-poor UHC. Along with each of these messages, we laid out two sets of recommendations for how the benefit could be achieved:

- A set of what we called ‘national opportunities’: policy proposals for national policymakers, particularly ministries of finance and health
- A set of ‘opportunities for international collective action’: policy proposals for the international community, particularly aid donors.

One advantage of this approach is that we were talking to specific audiences with specific policy messages, which was helpful when it came to post-publication outreach. Given that global health or development commissions are usually convened to help spur action on a pressing or neglected challenge, it is helpful to develop an action plan for the publication date and beyond. This plan includes identifying the most important policy makers that the commission wants to reach, the strategies and venues for reaching them, and the mechanisms for maximizing policy impact and dissemination. The plan that the CIH developed was based on using all of the resources available, from the commissioners themselves, who were early champions of the research and helped developed the recommendations, to key influencers in our networks who were committed to the CIH work.

We knew that busy policymakers would be very unlikely to read the full report, so we spent time producing a collection of highly digested briefing materials written in an accessible style that used colourful figures and graphs to bring the messages alive. We worked with a communications firm to produce three kinds of materials:

- Policy briefs: We created a series of five short, attractively designed two-page policy briefs that had targeted messages for targeted audiences. The five briefs were on: (1) key take-home messages of the report, (2) achieving a grand convergence, (3) curbing NCDs and injuries, (4) the returns to investing in health, (5) opportunities for low- and middle-income countries and (6) opportunities for the international community. We translated these into Arabic, French and Spanish.
- Slides: We produced a short slide deck about *Global Health 2035* that commissioners could use to give talks in their own countries.
- Video: We made a 3 min of video about grand convergence narrated by the CIH chair. We often used this video to kick off in-person briefings about the report.

We also had a press strategy in place, as we saw the press as a critical pathway to reach our intended policy actors. Recognizing that reporters’ interest would peak at the moment we launched, and around specific high profile moments during which the commissioners engaged with the content of the report, we carefully planned specific activities and moments to engage influential journalists. Our launch strategy included a press release, invitations to the media to attend the launches (we had simultaneous events in London, Tunis and Johannesburg), a press availability with the Commission chairs at the London launch event and a series of Op-Eds written by multiple commissioners in various newspapers. Importantly, the outreach activities did not stop with high profile launches in multiple geographies, and a detailed and ongoing calendar of events and moments were used to maximize the reach of the report findings and reinforce the messages. Different voices within the commission were also highlighted to reach different policy audiences depending on whether the goal was reaching a group of health or finance ministers or a set of high-level donors. The commissioners themselves were both the messengers to broader audiences and strong advocates in their own policy circles.

### Using the final report to lay the groundwork for future work

It is critical to ensure there is a clear end date so that commissioners know what is expected from them, what the intended outcome is, and what the next steps are for the commission findings. Having said this, the lifespan of a commission can be extended if it lays the foundation for a research agenda that is then taken up after the commission report is published.

The CIH’s *Global Health 2035* report planted a few seeds that later grew into fully fledged research studies. To give just one example, the report introduced the notion that the international donor community is neglecting the ‘core functions’ (also called ‘global functions’) of donor financing for health. These functions comprise the provision of global public goods (e.g. knowledge generation), the management of negative cross-border externalities (e.g. pandemics) and fostering global leadership and stewardship (e.g. improving aid effectiveness). In the 18 months after publication of *Global Health 2035*, we went on to conduct empirical research to quantify donor funding for these functions. Our study, published in *The Lancet* in December 2015, found that only one-fifth of all donors financing for health is channelled to these functions (Schäferhoff *et al.* 2015). This work in turn has led to the launch of a new Working Group on International Collective Action for Health, which is guiding a program of research led by Duke’s new Center for Policy Impact in Global Health (Center for Policy Impact in Global Health 2017). 

## Conclusion

In reflecting on our experience convening the CIH, we think that certain elements were important, including the novelty, simplicity and timing of the messaging; producing empirical research as part of the CIH process; making our recommendations forward-looking; getting the right chair and mix of commissioners and developing policy engagement and outreach plans that aimed at reaching a diverse set of policy relevant audiences through different channels (e.g. the media, personal networks and online and social media platforms). Perhaps most importantly, to quote Friedrich Nietzsche, we felt that our key task was ‘to make an individual uncomfortable’—that is, we wanted our commission report to stimulate debate and dissent on what kind of global health transformation is achievable in our lifetimes. The letters that *The Lancet* received in response to our report suggest that, in this one respect at least, we succeeded (Chiriboga *et al.* 2014; Fryatt 2014; Yates and Dhillon 2014; Yamey *et al.* 2014).
